# Metatranscriptomic and metagenomic description of the bacterial nitrogen metabolism in waste water wet oxidation effluents

**DOI:** 10.1016/j.heliyon.2017.e00427

**Published:** 2017-10-18

**Authors:** Julien Crovadore, Vice Soljan, Gautier Calmin, Romain Chablais, Bastien Cochard, François Lefort

**Affiliations:** a Plants and pathogens group, Institute Land Nature and Environment, Hepia, HES-SO University of Applied Sciences and Arts Western Switzerland, 150 route de Presinge, 1254 Jussy, Switzerland; b Puratis Sàrl, EPFL Innovation Park, Building C, 1015 Lausanne, Switzerland; c Faculty of Engineering and Architecture, HES-SO University of Applied Sciences and Arts Western Switzerland, Rue de la Jeunesse 1, 2800 Delémont, Switzerland

**Keywords:** Applied sciences, Biological sciences, Environmental science, Genetics, Microbiology

## Abstract

Anaerobic digestion is a common method for reducing the amount of sludge solids in used waters and enabling biogas production. The wet oxidation process (WOX) improves anaerobic digestion by converting carbon into methane through oxidation of organic compounds. WOX produces effluents rich in ammonia, which must be removed to maintain the activity of methanogens. Ammonia removal from WOX could be biologically operated by aerobic granules. To this end, granulation experiments were conducted in 2 bioreactors containing an activated sludge (AS). For the first time, the dynamics of the microbial community structure and the expression levels of 7 enzymes of the nitrogen metabolism in such active microbial communities were followed in regard to time by metagenomics and metatranscriptomics. It was shown that bacterial communities adapt to the wet oxidation effluent by increasing the expression level of the nitrogen metabolism, suggesting that these biological activities could be a less costly alternative for the elimination of ammonia, resulting in a reduction of the use of chemicals and energy consumption in sewage plants. This study reached a strong sequencing depth (from 4.4 to 7.6 Gb) and enlightened a yet unknown diversity of the microorganisms involved in the nitrogen pathway. Moreover, this approach revealed the abundance and expression levels of specialised enzymes involved in nitrification, denitrification, ammonification, dissimilatory nitrate reduction to ammonium (DNRA) and nitrogen fixation processes in AS.

## 1 Introduction

Municipal sludge management and disposal include dewatering, storage and transport to landfill; the costs of this process are increasing and this problem needs to be addressed. Sludge treatments represent 50% of the operating costs of wastewater treatment and are therefore a major target to save energy (Nowak, 2006). Anaerobic digestion is the most common method used to reduce the amount of final sludge solids and enable biogas production.

The wet oxidation (WOX) technology is one among others available as an environment-friendly alternative to waste sludge going to landfill. WOX allows for the elimination of organic components in the liquid phase by oxidation at high temperature and pressure (Baroutian et al., 2013). It can be set up as the sole installation or as a pre-treatment device to reduce sludge amounts. The final effluent, following the WOX process, can be returned to the wastewater treatment plant or introduced in anaerobic digesters. If the WOX is highly efficient in degrading complex organic compounds (Baroutian et al., 2015 and Prince-Pike et al., 2015), the resulting effluent is rich in low molecular weight organics, inorganic acids and inorganic salts, and contains highly concentrated ammonia (Genc et al., 2002). WOX is therefore used to improve anaerobic digestion, by maximising the conversion of carbon into methane, which represents an alternative to incineration, landfilling and dispersion of sewage sludge in farm fields. The weak point lies in the high ammonia concentration of the WOX effluent, since ammonia is known as a very powerful inhibitor of anaerobic processes, affecting the activity of methanogens, which are among the anaerobic microorganisms less tolerant to ammonia (Kayhanian, 1994). Kroeker et al. (1979) reported that an ammonia concentration as little as 1.7 g/L can reduce methane production, which means that using WOX as a pre-treatment step in anaerobic digestion, would produce adverse effects. In order to reduce the negative impact of ammonia on methanogens, a biological treatment using aerobic granular sludge has been evaluated in the course of this study. Producing aerobic granules is a promising technology for the biological treatment of high strength industrial wastewater (Song et al., 2015), because a granular biomass has a strong structure and excellent settling properties, compared to bioflocs. A granular sludge was first described for strictly anaerobic systems by Lettinga et al. (1984). The principle of this technology was then adapted and recently optimized for the formation and application of aerobic granules (Stuermer et al., 1982; Inizan et al., 2005; Shi et al., 2010; Cydzik-Kwiatkowska and Wojnowska-Baryła, 2011; Shi et al., 2011a; Li et al., 2012; Shen et al., 2013 and Derlon et al., 2016). A granular sludge has improved settling characteristics, operating the liquid separation of the solid**-**biomass very smoothly. Using aerobic granules results in handling smaller settlers (US Environmental Protection Agency, 2013; Adav et al., 2008). The compact structured aerobic sludge granules are characterised by a wide microbial diversity, high biomass retention and a high tolerance to toxicity (US Environmental Protection Agency, 2013). The granulation of microorganisms occurs under specific process conditions, made easier by the excretion of microbial biopolymers that are used for microgranulation of selected microorganisms. In further stages, microgranules are merged into granules (Liu et al., 2004; Zhang et al., 2007). The processes analysed in the present study are part of a development project for a biological process, using aerobic granules to remove high ammonia concentration from the WOX, and addressing the major drawbacks of existing technologies such as price, selectivity, stability, sensitivity and process efficiency.

Metagenomics and metatranscriptomics, respectively DNA and mRNA sequence based, recently became the tools of choice for deciphering bacterial taxonomy and protein encoding genes, in complex environmental samples like activated sludge (Yu and Zhang (2012); Muller et al. (2014); Law et al. (2016)), soil (Tveit et al., 2014), marine environment (Shi et al., 2011b; Kopf et al., 2015) or human gut (Franzosa et al., 2014). In this study, we applied these tools to analyse the dynamics of activated sludge microbial communities in bioreactors, and their respective and relative expression levels of nitrogen removal genes, in order to improve large-scale water treatment processes.

## 2 Materials and methods

### 2.1 Experiments in bioreactors

**Fig. 1:**
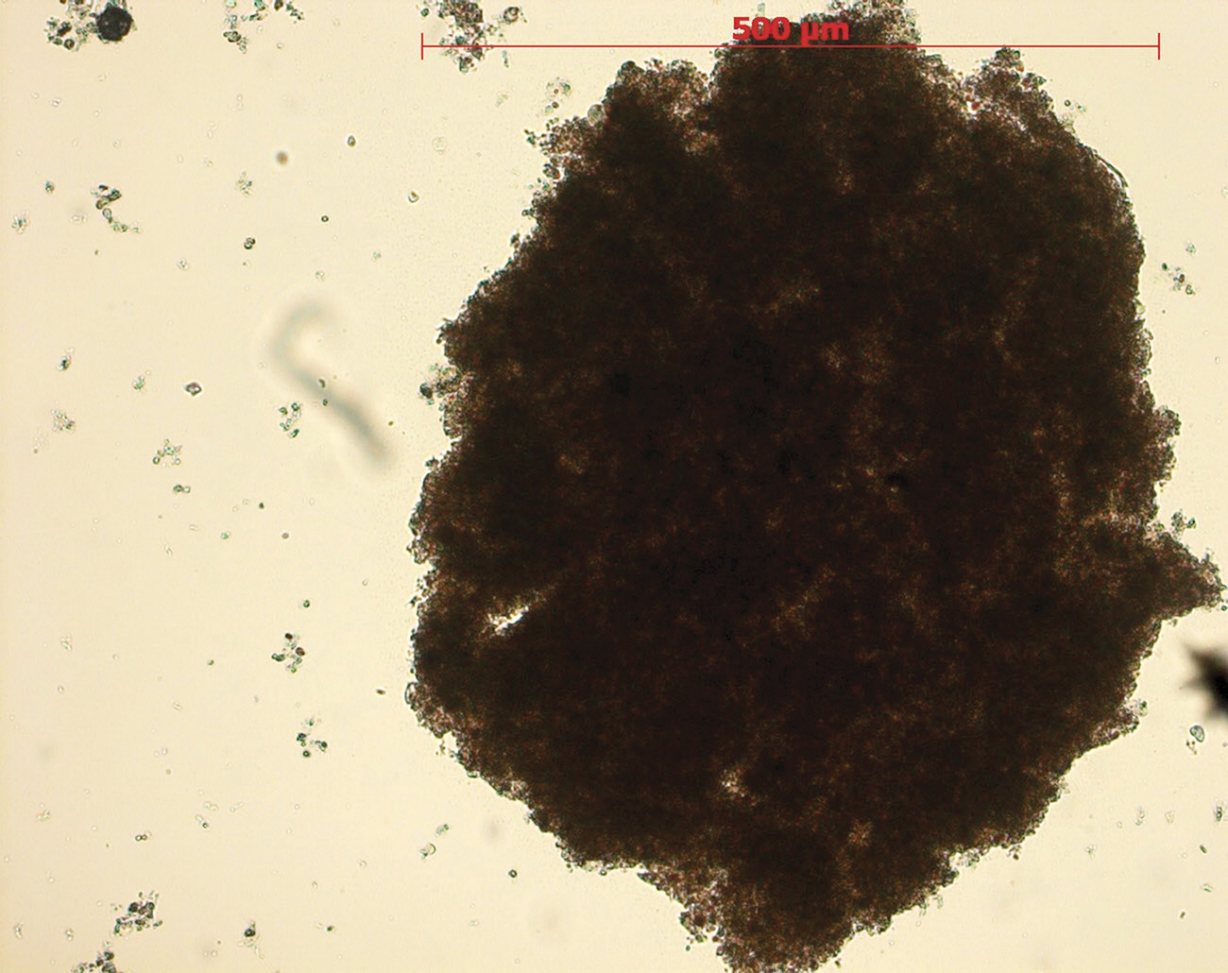
Microscope observation of one Aerobic Granule formed after three months from pre-adapted Activated Sludge on a Wet Oxidation effluent (bar length = 500 μm).

Two different experiments were set up in aerated glass reaction vessels inoculated with 5 L of activated sludge (AS), so as to obtain a starting Mixed Liquor Suspended Solids (MLSS) concentration of 5 g/L. The MLSS is measured on samples, dried for 2 h at 105 °C. WTW pH electrodes were used to monitor pH, temperature and concentration of dissolved oxygen. Aeration was performed with compressed air. Fine air bubbles for aeration were introduced through an air diffuser in the bottom of bioreactors. The first experiment in bioreactor 1 (B1) was inoculated with a pre-adapted AS (on diluted WOX effluents) from the wastewater treatment plant of Rovereto, Italy. The second experiment in bioreactor 2 (B2) was inoculated with the same pre-adapted AS, to which were added 200 mL of granular biomass (Aerobic Granules, AG). Microscope observations of the granular biomass were carried out with a dissecting microscope Axiostar (Zeiss, Germany) mounted with an Axiocam ERc5s camera (Zeiss). Fig. 1 shows a sample of the granular biomass.

The granulation process of the adapted activated sludge using mixtures of wet oxidation effluents and landfill leachate, as a substrate, was followed. Both experiments were run in parallel for one month, with a four-step cycle (substrate addition, aeration, sedimentation and water decantation), repeated every 3 days. The granulation process of the activated sludge in the reaction vessels was followed through microscopic observation and chemical parameters. The substrate consisted of a 1 L WOX mixture from Orbe waste water treatment plant (SWITZERLAND) at a concentration of 400 mg/L ammonia, plus leachate and water. The WOX samples are chemically described in Tables 1, 2 and 3. Table 1 gives the chemical composition of WOX samples, while Table 2 shows the major compounds present in the WOX effluent and Table 3, the solvents in minor concentration. The final concentration of ammonia was in the range 250**–**300 mg/L. The pH value was maintained between 6 and 8 throughout the experiment.

**Table 1:** Chemical composition of the WOX effluent samples.

Type	NH4-N mg/L	COD mg/L	pH
WOX 1	3800	12110	7.78
WOX 2	5100	12625	7.76
WOX 3	4350	13035	7.66
WOX 4	4200	12845	7.77
WOX 5	4250	12630	7.78
WOX 6	3750	13350	8.05
WOX 7	4200	12945	7.89

**Table 2:** Volatile fatty acids (VFA) present in the wet oxidation effluent.

Compounds	Units	Sample 1	Sample 2
Acetic acid	mg/l	3324.29	3489.58
Propionic acid	mg/l	129.32	135.33
Isobutyric acid	mg/l	99.09	107.88
Butyric acid	mg/l	12.67	13.41
Isovaleric acid	mg/l	17.18	18.43
Valeric acid	mg/l	7.64	7.85

**Table 3:** Solvents present in the WOX effluent.

Solvents	Units	Average values
Methanol	mg/l	135
Methyl ethyl ketone	mg/l	17
Methyl isobutyl ketone	mg/l	0
Ethanol	mg/l	6
n-buthanol	mg/l	0
isopropanol + acetone	mg/l	132

### 2.2 Analytical procedures

The chemical oxygen demand (COD) of the samples and the changes in N-NH_4_ and N-NO_3_ concentrations were determined using commercial Merck tube kits, which were analysed on Spectral photometer VEGA 400 (Merk, Germany). Biomass of the mixed microbial cultures was determined gravimetrically, by filtering the samples through a 0.45 μm membrane and drying it over 8–10 h at 110 °C.

### 2.3 Samples collection

Samples were collected from the bioreactors in 50 mL Falcon RNase and DNase free tubes for the 2 initial samples (Initial Activated Sludge (IAS) and Initial Activated Sludge + Aerobic Granules (IAS + AG)) and one month later for the 2 final samples (Final Activated Sludge (FAS) and Final Activated Sludge + Aerobic Granules (FAS + AG)). Samples were instantly frozen in liquid nitrogen containers prior to nucleic acids extraction. Sample duplicates were stored at −80 °C.

### 2.4 DNA and RNA extraction, library preparation and sequencing

**Fig. 2:**
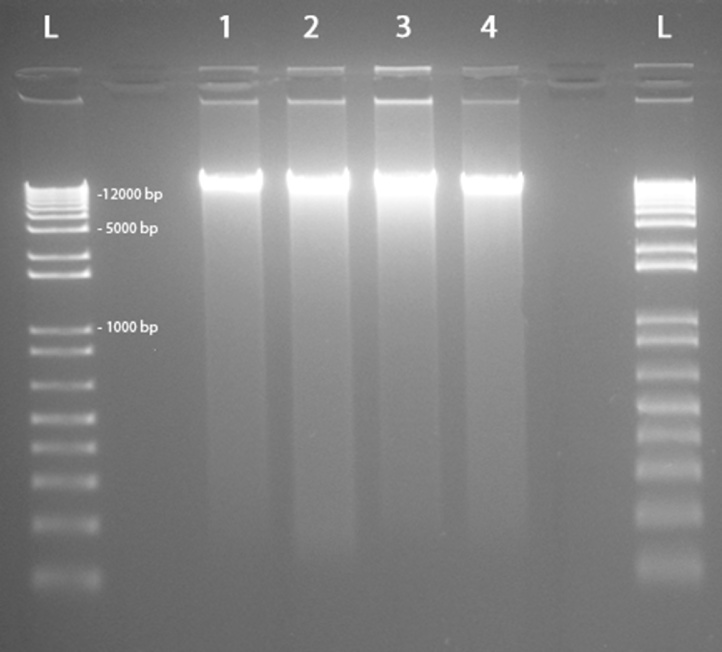
Quality control of DNA samples evaluated through gel electrophoresis.L: Ladder; 1. IAS-DNA; 2. FAS-DNA; 3. IAS + AG DNA; FAS + AG DNA.

**Fig. 3:**
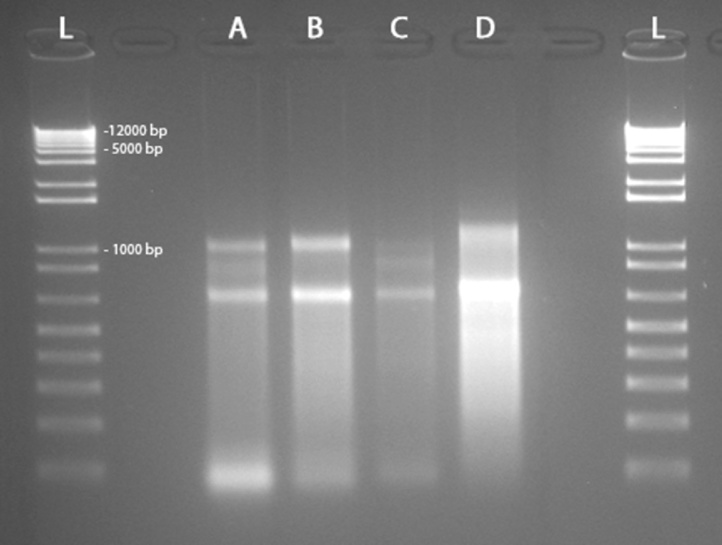
Quality control of RNA samples evaluated through gel electrophoresis. L: Ladder; 1. IAS-RNA; 2. FAS-RNA; 3. IAS + AG RNA; FAS + AG RNA.

Total nucleic acids were extracted from the 4 samples within 3 h after sampling. Raw samples were quickly thawed and centrifuged 8 min at 18,000 rpm at a temperature of 4 °C, and 250 mg of each pellet were then collected in triplicates. DNA extraction was performed with the PowerMax^®^ Soil DNA Isolation Kit (MO-BIO Laboratories, Inc., CA, USA) while RNAs were purified with Powersoil^®^ Total RNA Isolation Kit (MO-BIO Laboratories). RNA purification was continued by a DNase treatment with the Amplification Grade DNase I Kit (Sigma-Aldrich, Inc., MO, USA), in order to remove genomic DNA traces. DNA and RNA integrity was finally checked with electrophoresis gel (Fig. 2 and Fig. 3), and the quality and concentration of nucleic acids were assessed with the Nanodrop ND1000 spectrophotometer (NanoDrop Technologies, Thermo Fisher Scientific Inc., Switzerland). Metagenomic libraries were constructed using the Illumina TruSeq Nano DNA Preparation kit (Illumina, CA, USA). Prior to the construction of metatranscriptomic libraries, prokaryotes and eukaryotes rRNA were depleted using the MICROBExpress™ kit (Ambion, Thermo Fisher Scientific Inc., Switzerland). cDNA libraries were prepared with the Illumina TruSeq Stranded mRNA Sample Preparation kit. Finally, all libraries were multiplexed and sequenced in one single and full paired-end reads lane (2 × 100 bp) of an Illumina Hiseq2000 High Output run. The FASTQ files for all paired-end libraries were imported into the FastQC software (http://www.bioinformatics.bbsrc.ac.uk/projects/fastqc) to perform a quality control of the raw sequencing datasets. The removal of index and adapter, as well as the reads and quality trimming were completed with Trimmomatic version 0.27 (Bolger et al., 2014). The bases with a quality score ≥ 30 were retained by applying a 4 bp sliding window, and only the complete read pairs were kept.

### 2.5 Bioinformatic analysis

All bioinformatics analyses, for taxonomy and identification of genes of the nitrogen metabolism, were performed on the MG-RAST (MetaGenomics Rapid Annotation using Subsystem Technology) pipeline (Meyer et al., 2008), dedicated to metagenomes analysis.

**Fig. 4:**
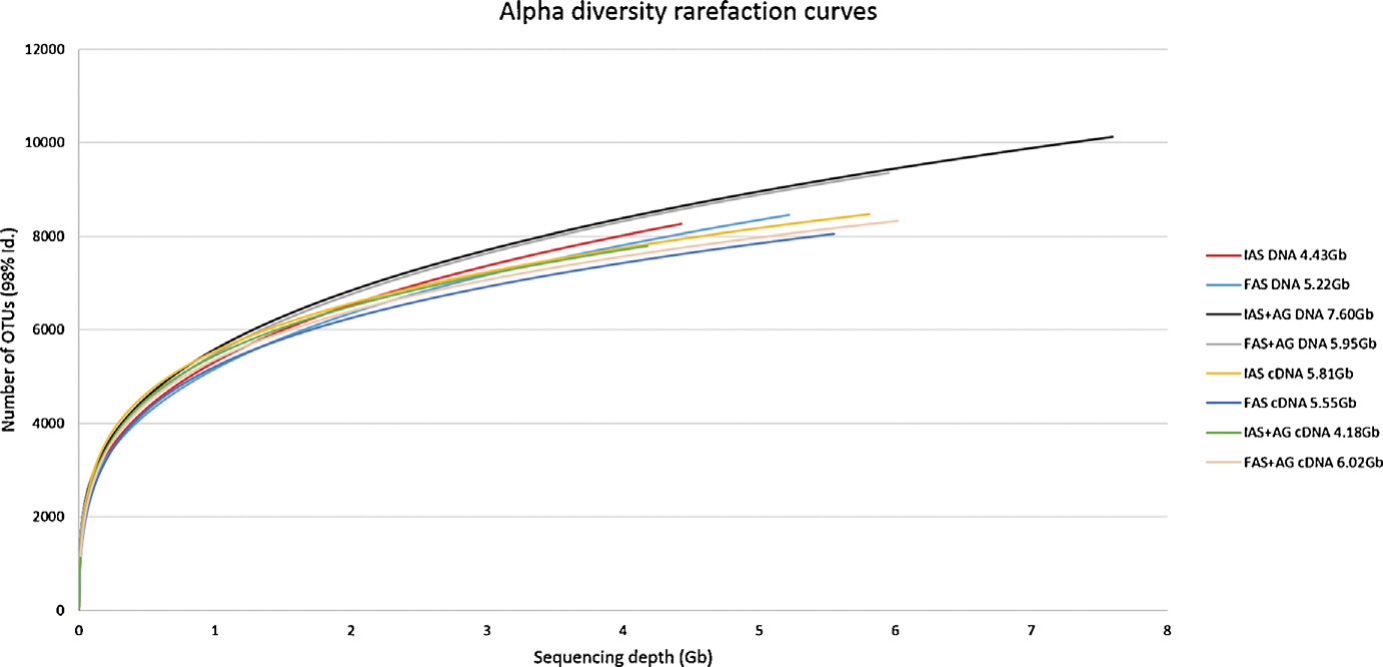
Alpha diversity rarefaction curves of the microbial richness of the 8 samples according to their respective sequencing depth, sampling date (Initial (I) or Final (F) sampling), nature (Activated Sludge (AS) or AS plus Aerobic Granules (AG)) and datasets (DNA or cDNA). OTUs were computed from the M5NR protein database (accessed through MG-RAST) using a maximum e-value of 1e-5, a minimum identity of 98%, and a minimum alignment length of 15 measured in amino acids for protein (including Bacteria, Archaea, Eukaryota, and Viruses).

In order to assess the annotated species richness of the eight samples and the influence of the sequencing depth (from 4.4 to 7.6 Gb), alpha diversity rarefaction curves (Fig. 4) were computed based on MG-RAST with the M5NR protein database using a maximum e-value of 1e-5, a minimum identity of 98%, and a minimum alignment length of 15 measured in amino acids for protein (including Bacteria, Archaea, Eukaryota, and Viruses). M5NR (Non-Redundant Multi-Source Protein Annotation Database) is an integration of many sequence databases (SEED, KEGG, NCBI, UniProt etc.).

OTU clustering was computed with the Blast-Like-Alignment Tool (BLAT) (Kent, 2002) from the M5NR protein database (accessed through MG-RAST), using a maximum e-value of 1e-5, a minimum identity of 98%, and a minimum alignment length of 15 measured in amino acids for protein. We used MG-RAST default clustering parameters within the BLAT algorithm.

The bioinformatics analyses were carried out according to the method proposed by Yu and Zhang (2012) with the same selection thresholds.

To allow the comparison between the 8 samples, equal sub-datasets of 4 Gb were randomly generated with the software mothur 1.35.1. (Schloss et al., 2009), using the sub.sample command line: mothur > sub.sample(filename.fasta,size = lowestnumber).

The metatranscriptomics data were compared to M5NR in MG-RAST, using a maximum e-value of 1e-5, a minimum identity of 85%, and a minimum alignment length of 15 measured in amino acids for protein and base pairs for RNA databases. The generated results were visualized using the tools of the Krona hierarchical data browser (Ondov et al., 2011).

DNA and cDNA nucleotide sequences libraries were submitted and deposited at the MG-RAST database (accessible at http://metagenomics.anl.gov/) and made publicly available under the accession numbers 4568625.3 and 4568626.3 for IAS_DNA_ (respectively R1 and R2 Illumina paired-end reads files each time), 4568713.3 and 4568714.3 for the IAS_cDNA_, 4572171.3 and 4572172.3 for the FAS_DNA_, 4569604.3 and 4569605.3 for the FAS_cDNA_, 4569569.3 and 4569570.3 for the IAS + AG_DNA_, 4572294.3 and 4572295.3 for the IAS + AG_cDNA_, 4569598.3 and 4569599.3 for the FAS + AG_DNA_, and finally 4572296.3 and 4572297.3 for the FAS + AG_cDNA_. Metagenomes and metatranscriptomes raw sequencing datasets have also been made publicly available from the Sequence Read Archive (SRA) (Leinonen et al., 2011) of the National Center for Biotechnology Information (NCBI, accessible at http://www.ncbi.nlm.nih.gov/Traces/sra) under the following SRA accession numbers: SRP050327 for the IAS_DNA_, SRP051963 for the FAS_DNA_, SRP052005 for the IAS + AG_DNA_, SRP052006 for the FAS + AG_DNA_, SRP052007 for the IAS_cDNA_, SRP052008 for the FAS_cDNA_, SRP052009 for the IAS + AG_cDNA_ and SRP052010 for the FAS + AG_cDNA_ (Crovadore et al., 2017).

## 3 Results and discussion

### 3.1 Metagenomes and metatranscriptomes characteristics

The high throughput sequencing of the 8 multiplexed samples, using an Illumina Hiseq 2000 single and full paired-end reads lane, allowed to generate genomics information on AS metabolisms and its active microorganisms, unreached so far. As shown in Table 4 and Table 5, the four raw and combined metagenomes datasets yielded between 4.428 Gbp and 7.599 Gbp, while the four raw and combined metatranscriptomes yielded between 4.177 Gbp and 6.017 Gbp. All data were submitted to analyses in MG-RAST and processed through the quality control (QC) pipeline. The resulting sequencing depths of metagenomes ranged from 4.064 to 7.599 Gbp (with QC failed reads from 4.65 to 8.20%), and the ones of metatranscriptomes ranged from 4.001 to 5.833 Gbp (with a smaller QC failed reads from only 2.60 to 4.20% compared to metagenomic reads). Finally, equal sub-datasets of 4 Gbp were randomly generated to allow comparison between DNA or cDNA experiments, between initial and final sampling results or between the 2 different bioreactors at the same sequencing depth.

**Table 4:** Metagenomes features based on the percentage of reads assigned. (accn: accession; Mb: Million bases; QC: Quality Control; rRNA: ribosomal RNA; Sd: Sequencing depth; R1 and R2 stand for forward and reverse Illumina paired end reads).

Metagenomes	IAS_DNA_		FAS_DNA_		IAS + AG_DNA_		FAS + AG_DNA_	
Library codes	GCZ12_R1	GCZ12_R2	GCZ13_R1	GCZ13_R2	GCZ14_R1	GCZ14_R2	GCZ15_R1	GCZ-15 R2
MG-RAST accn	4568625.3	4568626.3	4572171.3	4572172.3	4569569.3	4569570.3	4569598.3	4569599.3
Sequencing depth (Mb)_Upload	4'428 Mb		5'221 Mb		7'599 Mb		5'952 Mb	
Failed MG-RAST QC pipeline (%)	12.20%		5.05%		5.55%		4.65%	
rRNA	2.06%		1.65%		3.10%		3.10%	
Annotated Protein	27.60%		31.65%		40.65%		42.95%	
Predicted but unknown Protein	49.70%		52.60%		43.20%		42.2%	
Unknown	8.55%		9.05%		7.50%		7.15%	
MG-RAST post QC Sd_Processed	3.887 Mb		4'957 Mb		7'177 Mb		5.675 Mb

**Table 5:** Metatranscriptomes features based on the percentage of reads assigned. (accn: accession; Mb: Million bases; QC: Quality Control; rRNA: ribosomal RNA; Sd: Sequencing depth; R1 and R2 stand for forward and reverse Illumina paired end reads).

Metatranscriptomes	IAS_cDNA_		FAS_cDNA_		IAS + AG_cDNA_		FAS + AG_cDNA_	
Library codes	GCZ16_R1	GCZ16_R2	GCZ17_R1	GCZ17_R2	GCZ18_R1	GCZ18_R2	GCZ19_R1	GCZ19_R2
MG-RAST accn	4568713.3	4568714.3	4569604.3	4569605.3	4572294.3	4572295.3	4572296.3	4572297.3
Sequencing depth (Mb)_Upload	5'808 Mb		5'548 Mb		4'177 Mb		6'017 Mb	
Failed MG-RAST QC pipeline (%)	3.85%		2.60%		4,20%		3.05%	
rRNA	38.90%		38.65%		39,90%		37.65%	
Annotated Protein	57.25%		58.75%		55,80%		59.30%	
Predicted but unknown Protein	0%		0%		0%		0%	
Unknown	0%		0%		0%		0%	
MG-RAST post QC Sd_Processed	5'584 Mb		5'403 Mb		4'001 Mb		5'833 Mb	

The annotation of the metagenomes sequences datasets, as shown in Table 4, revealed the presence of a small amount of rRNA coding reads (from 1.65 to 3.10%) compared to protein coding reads, which prevailed at around 80% (addition of both “annotated” and “predicted but unknown” proteins). Looking more closely at the allocated identities of protein coding reads, more than half of these sequences remained “predicted but unknown” in all samples. On the other hand, as shown in Table 5, the annotation of the metatranscriptomes indicated a higher amount of “rRNA” coding reads (from 37.65 to 39.90%), despite use of the rRNA depletion step before the library preparation, and a higher percentage of “annotated proteins” (from 55.80 to 59.30%) without any “predicted but unknown protein” fraction. As shown in Table 4 and Table 5, “unknown” sequences averaged at 8.06% for the 4 metagenomes, while they were marginal in the 4 metatranscriptomes (MG-RAST classifies reads that “do not contain any recognized feature” in this “unknown” category). The annotation results of metagenomics and metatranscriptomics were very similar and reproducible between the samples of each approach, but metatranscriptomics appeared to provide a different characterisation of samples, since all sequences had been assigned to an identity.

### 3.2 Species diversity of microbial communities

The Alpha diversity rarefaction curves of the eight DNA and cDNA raw datasets (Fig. 4), based on OTUs with a minimum identity of 98%, showed that even the highest sequencing depth of 7.6 Gb, yielding 10134 unique species did not allow to reach the asymptote. However, the rarefaction analysis indicated that, as the rarefaction curves were approaching their respective plateau, all libraries represented the microbial communities well. For instance, the rarefaction analysis of IAS + AG metatranscriptomic sample (black curve) yielded 8397 species at a 0 − 4 Gb sequencing depth, and 10134 species at 7.6 Gb, which means that the supplementary 3.6 Gb sequencing depth only yielded 1737 additional species. It appeared therefore that a 4 Gb sequencing depth would yield enough information for all samples of the present study.

As shown in Fig. 4, at a common 4 Gb sequencing depth, metagenomic analysis revealed a larger species richness, from a minimum 7816 OTUs to a maximum 8397 OTUs, whereas a metatranscriptomic analysis, which only targeted living microorganisms, revealed a minimum 7441 to a maximum 7750 OTUs. At the common 4 Gb sequencing depth, AS + AG initial and final samples all displayed a larger species richness when compared to AS initial and final samples, which can be viewed as a consequence of the added Aerobic Granules microbial richness. Interestingly, the rarefaction analyses showed, for metagenomics as well as for metatranscriptomics, that final samples for both AS and AS + AG were always characterised by a lower species richness than the one observed in their respective initial samples, which suggests that, depending of the substrate and culture conditions, microbial selection could have occurred.

### 3.3 Taxonomic domains proportions

**Fig. 5:**
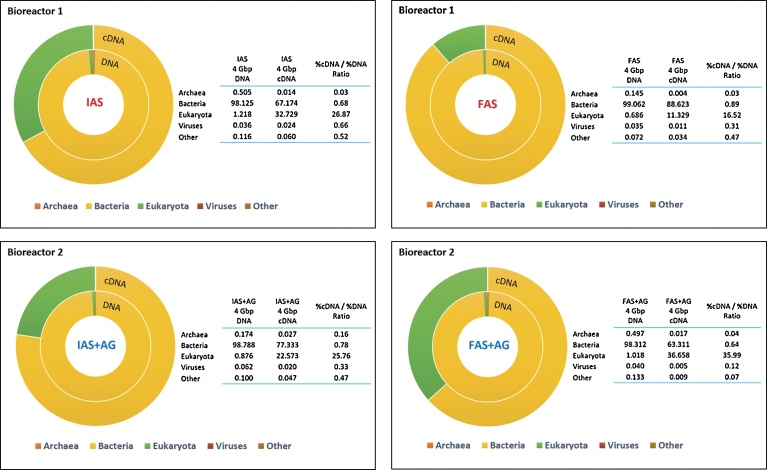
Taxonomic domain proportions in Bioreactors 1 and 2 from the initial to the final state, based on either total DNA or cDNA raw sequencing datasets. Domains are represented by Archaea, Bacteria, Eukaryota, Viruses and Other. IAS and FAS stand for respectively Initial and Final Activated Sludge and characterize the Bioreactor 1. IAS + AG and FAS + AG stand for respectively Initial and Final Activated Sludge plus Aerobic Granules and characterize the Bioreactor 2.

As summarised in Fig. 5, at the beginning and at the end of the experiment one month later, bacteria were the main represented domain for the two different bioreactor samples in both DNA and cDNA datasets. More precisely and considering the 4 different samples in this order: IAS, FAS, IAS + AG and finally FAS + AG, the bacteria domain accounted for 98.12%, 99.06%, 98.78% and 98.31%, respectively, of the total DNA sequences, while eukaryota represented only 1.21%, 0.68%, 0.87% and 1.01% respectively. Concerning total cDNA sequences, the bacteria domain represented 67.17%, 88.62%, 77.33% and 63.31% respectively, while the eukaryota sequences amounted to 32.72%, 11.32%, 22.57% and 36.65% respectively.

The Archaebacteria domain only ranged from a minimum 0.145% to a maximum 0.505% in total DNA sequences, and from a minimum 0.004% to a maximum 0.027% in total cDNA sequences. Viruses represented between 0.035% and 0.062% of total DNA sequences, and between 0.005% and 0.024% of total cDNA sequences.

As metatranscriptomics only target living microorganisms, Yu and Zhang (2012) determined that the relative activity of a microbial population could be defined as the ratio of its abundance/percentage in the cDNA dataset over its abundance/percentage in the DNA dataset. As described in Fig. 5, the %_cDNA_/%_DNA_ ratios were always inferior to 1 for the archaea, bacteria and viruses domains, whatever the samples. On the contrary, eukaryota %_cDNA_/%_DNA_ ratios increased and accounted for a minimum 16.52% to a maximum 35.99%. These results corroborate those obtained by Yu and Zhang (2012) with an activated sludge sample. Despite the shorter half-lives of most bacterial mRNAs, which usually range from 40 s to 60 min, bacteria sequences prevailed in both metagenomic and metatranscriptomic datasets. The fact that some eukaryotic mRNAs may have half**-**lives lasting several days could be the main explanation for the high eukaryotic %_cDNA_/%_DNA_ ratios that were observed (Richards et al., 2008; Belasco and Brawerman, 1993). Another explanation could be that some eukaryotic microorganisms benefited of the growth conditions of the bioreactors.

Finally, by looking more closely at the differences between initial and final samples, it can be noticed that the initial eukaryota %_cDNA_/%_DNA_ ratios (IAS and IAS + AG samples), 26.87% and 25.76% respectively, are relatively close. The eukaryota %_cDNA_/%_DNA_ ratio of the FAS sample decreased by 10.35%, while the eukaryota %_cDNA_/%_DNA_ ratio of the FAS + AG sample increased by 10.23%. This difference may result from a bias in the preparation step of the sequencing library, or from the expression of some eukaryote feeding on bacteria in the FAS + AG samples.

### 3.4 Identification and dynamics of the active microbial community at the genus level

**Fig. 6:**
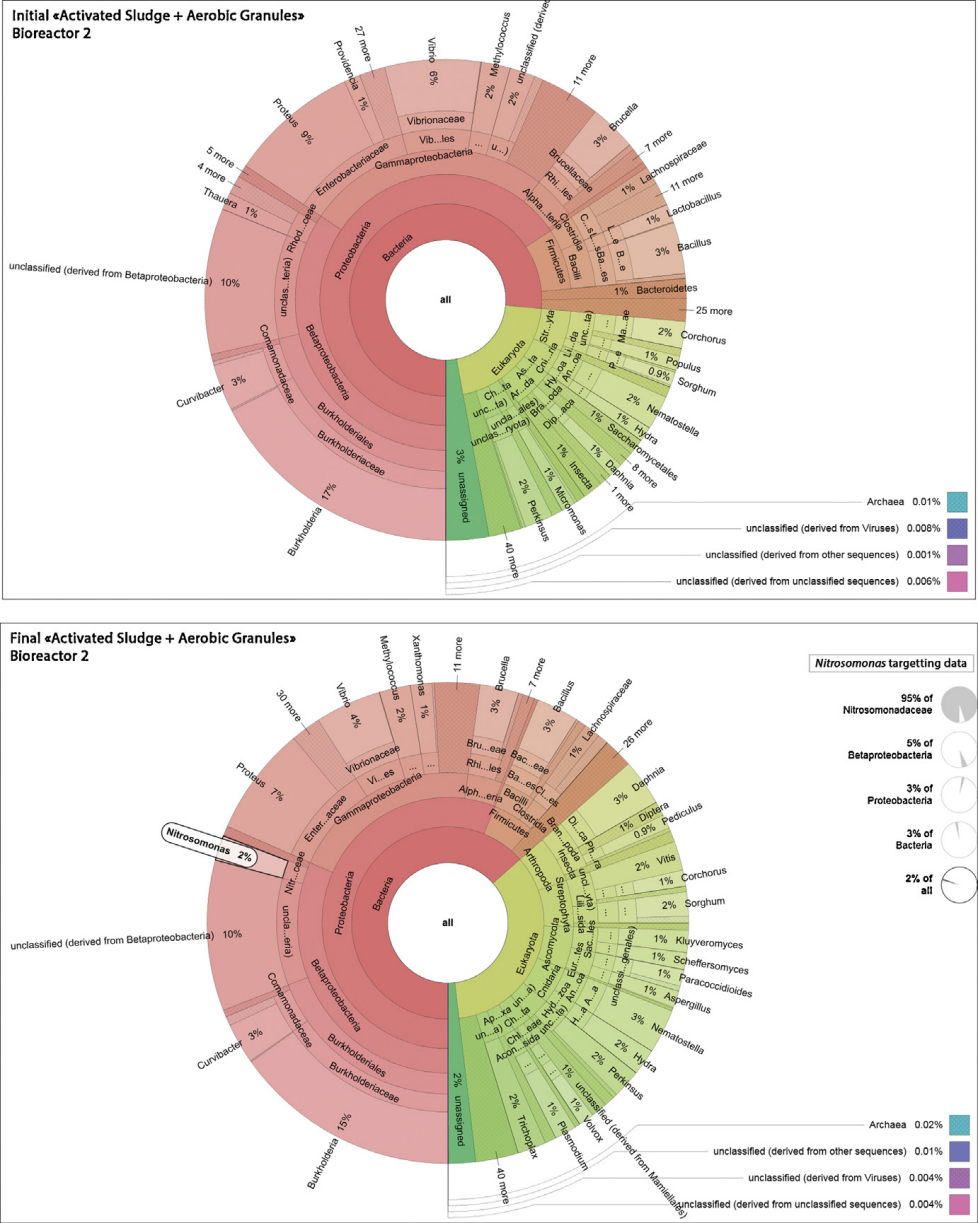
Metatranscriptomics taxonomy of Bioreactor 1, Activated Sludge: Adapted KRONA chart of microbial communities identified at the genus level (based on total cDNA raw sequencing datasets) in Bioreactor 1 at the initial and final stages. The genus *Nitrosomonas* is highlighted.

**Fig. 7:**
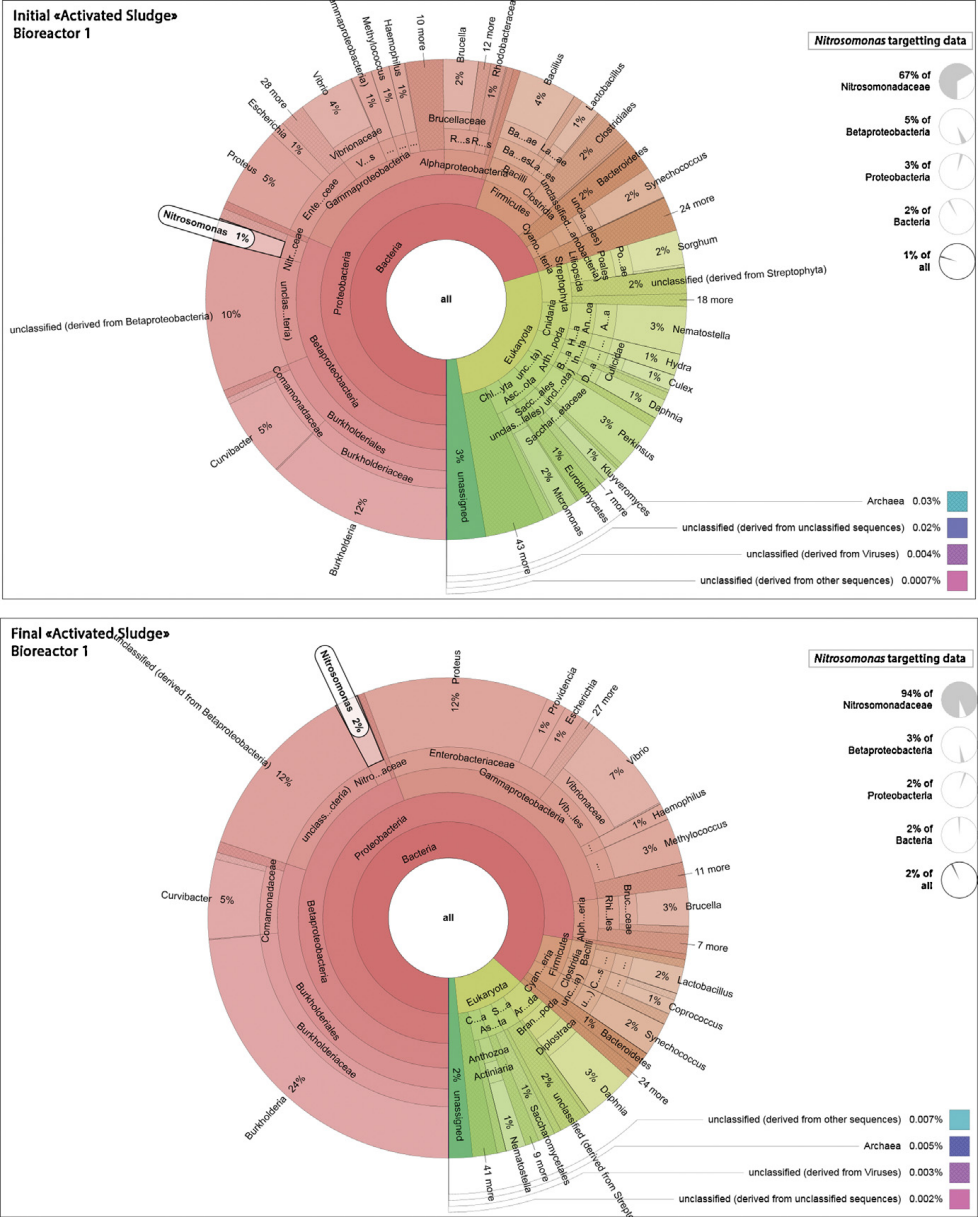
Metatranscriptomics taxonomy of Bioreactor 2, Activated Sludge + Aerobic Granules: Adapted KRONA chart of microbial communities identified at the genus level (based on total cDNA raw sequencing datasets) in Bioreactor 2 at the initial and final stages. The genus *Nitrosomonas* is highlighted.

The metatranscriptomics analyses reflect the diversity of active microorganisms more accurately than would do metagenomics, which is based on DNA from living but also dead cells. Fig. 6 and Fig. 7 show how the identities of microorganisms are distributed at the genus level, in the 4 metatranscriptomes provided by the M5NR annotation database from the MG-RAST pipeline. The *Nitrosomonas* genus, which is important in the nitrogen cycle by oxidising ammonia into nitrites, has been highlighted.

As described in these 4 circular diagrams (Fig. 6 and Fig. 7), both activated sludge bioreactors, that were fed a WOX mixture with a high ammonia concentration for over one month, are characterised by a huge diversity of living microorganisms at the genus level, some of them prevailing. The most represented genus in all samples, i.e. IAS and FAS from B1, IAS + AG and FAS + AG from B2, was the Betaproteobacterial *Burkholderia* genus, accounting for 12%, 24%, 17% and 15% respectively of all sequences. This genus encompasses soil species known as activated sludge xenobiotic biodegraders (Yang et al., 2016; Zhang et al., 2013), plant growth promoting rhizobacteria (PGPR) with nitrogenase activity (Gao et al., 2015), or as plant and opportunistic human pathogens (Coenye and Vandamme, 2003; Correia et al., 2008). Following *Burkholderia*, three other bacterial genera, *Proteus*, *Vibrio* and *Curvibacter,* accounted for over 3% of the total living microorganisms in all 4 samples. Other bacterial genera, well known as active species in sewage and activated sludge also appear in Fig. 6 and Fig. 7, including *Nitrosomonas*, *Methylococcus*, *Brucella* or *Lactobacillus,* for example. The layout of these 4 diagrams at the genus level, however, does not allow the predominant bacterial genera to be highlighted, i.e. those that would characterise the contribution of aerobic granules in IAS + AG and FAS + AG when compared to the samples of activated sludge. These figures illustrate the dynamics of these key bacterial genera during the experiment, For instance, Fig. 6 and Fig. 7 show that the *Nitrosomonas* proportion of all living microorganisms increased from 1% at IAS in B1 to 2% in FAS, and from less than 1% to 2% from IAS + AG to FAS + AG in B2. This may, in part, result from feeding of bioreactors a WOX mixture with high ammonia concentration, which promotes the nitrifiers (see Material and Methods). It is interesting to note that when compared to the Betaproteobacteria fraction exclusively, the percentage of *Nitrosomonas* sequences decreased from 5% to 3% between the initial and final samplings in B1 and, on the contrary, increased from less than 1% to 5% between initial and final sampling in B2. This reveals a fast and perpetual dynamics of adaptation of the bacterial communities to their environment.

Fig. 6 and Fig. 7 also disclosed an important genera diversity in the activated sludge samples, representing all the most important lineages. Metatranscriptomic analysis surprisingly showed that the half-lives of mRNA could be longer than ever expected. Indeed, such plant cDNAs were sequenced in the 4 activated sludge samples, and belong to *Sorghum*, *Vitis* or *Populus* genera, among others. A possible pollen contamination of the bioreactors from air is impossible, since sampling took place between early March and early April. Another interesting observation is the important growth of a *Daphnia pulex* population in both bioreactors. Daphnia is a genus of small planktonic crustaceans. Indeed, *Daphnia* mRNA reads increased from 1% to 3% in both bioreactors between the initial and final sampling. Belonging to the Cladocera order, the *Daphnia* species have been reported as living in many aquatic ecosystems from freshwater to swamps, and is a well-studied organism for bioassay in environmental tests (Chen et al., 2015a), or for its filtration abilities (Pau et al., 2013). Besides that, Fig. 6 and Fig. 7 also highlight the presence of many other prominent microorganisms such as the microalgae *Micromonas* genus, the fungus *Aspergillus*, or even the *Perkinsus* protist genus, unlike metatranscriptomics revealing that Archaeabacteria and viruses appeared as very minor and accounted for 0.03% and 0.008% of all microorganisms, respectively, in all samples.

### 3.5 Overall gene expression analysis and evolution

**Fig. 8:**
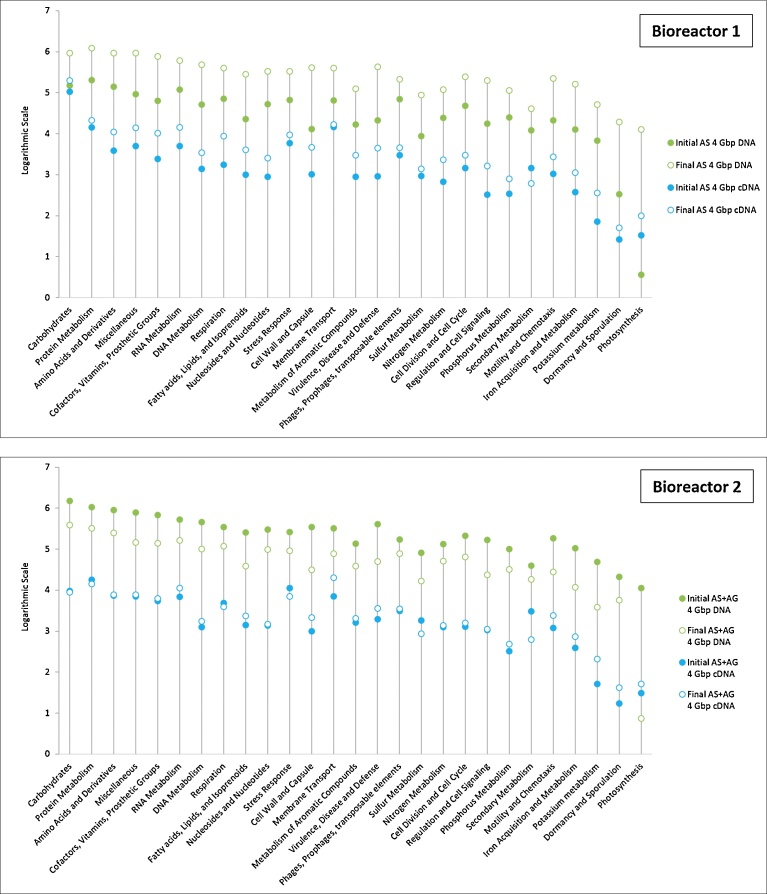
Comparison of global genes presence and expression between metabolic systems revealed by metagenomics and metatranscriptomics in the Bioreactors 1 and 2, at the initial and final stages (datasets extracted from MG-RAST SEED level 1 subsystem). Y-Axis scale stands for the logarithmic number of the sequences abundance (annotated sequences) per each category.

The analysis of the same samples, by metagenomics and metatranscriptomics, confered the advantage of allowing the evaluation of their relative gene expression (mRNAs) at a specific sampling time and its comparison with the potential gene profile (DNA) of these microbial communities. This can be used both at the taxonomy level and the global metabolic level, as described in Fig. 8 (Carbohydrates, Protein metabolism.) where 27 metabolisms are shown. In Fig. 8, the 8 equal generated sub-datasets of 4 Gbp, representing 1397904, 10159406, 174869, 347066, 9539499, 2390989, 99226 and 113412 sequences for IAS_DNA_, FAS_DNA,_ IAS_cDNA,_ FAS_cDNA,_ IAS + AG_DNA_, FAS + AG_DNA,_ IAS + AG_cDNA,_ FAS + AG _cDNA_, respectively, have been annotated with SEED level 1 Subsystem of MG-RAST. These results show that, at the same sequencing depth, the abundance of annotated genes in DNA datasets (green points in Fig. 8) is generally higher by 1 to 2 units (based on logarithmic scale) than their abundance in cDNA datasets (blue points in Fig. 8), confirming previous results obtained by Yu and Zhang (2012). In agreement with the results of several analyses on microbial communities of activated sludge (Yu and Zhang, 2012), soil (Urich et al., 2008) or marine environment (Gilbert et al., 2008), the results displayed in Fig. 8 show that the main metagenomic categories of gene sequences, expressed in the metabolism of the activated sludge samples of this study, are the “protein metabolism”, the “carbohydrates” and the “amino acids and their derivatives” systems, corresponding to the central metabolism. The carbohydrates genes prevailed in the sample AS + AG. Despite a higher protein metabolism potential, the expression of carbohydrate metabolism genes was by far the highest one in bioreactor 1. The expression of carbohydrate metabolism genes, accounted for 60.7% and 57.8% in the IAS and FAS cDNA datasets, respectively, against 9.6% and 7.8% in the IAS + AG and FAS + AG cDNA datasets of bioreactor 2. This shows a dramatic change of the metabolic processes induced by adding Aerobic Granules to the Activated Sludge. Then, in decreasing order of most expressed metabolisms in both bioreactors, came the metabolism of proteins and, more surprisingly, the membrane transport system, followed by or equal to amino acids and stress response. Finally, as highlighted in Fig. 8, after one month and at the same sequencing depth, the FAS DNA dataset was generally larger by one unit than the IAS DNA dataset in B1, while it was the contrary in the second bioreactor, where the FAS + AG dataset was smaller than the IAS + AG dataset. On the other hand, the final cDNA datasets were almost always larger than the initial cDNA datasets for both bioreactors. The metatranscriptomic analysis is not ambiguous, compared to metagenomics, and allows an accurate assessment of the expression of genes of the microbial community, over time and in function of their environment. This increase in overall metabolic activity in both bioreactors could be the result of an adaptation phenomenon, improving the efficiency of microbial communities in developing from a given media (substrate, pH, temperature, oxygen, etc.) (Chen et al., 2015b).

**Fig. 9:**
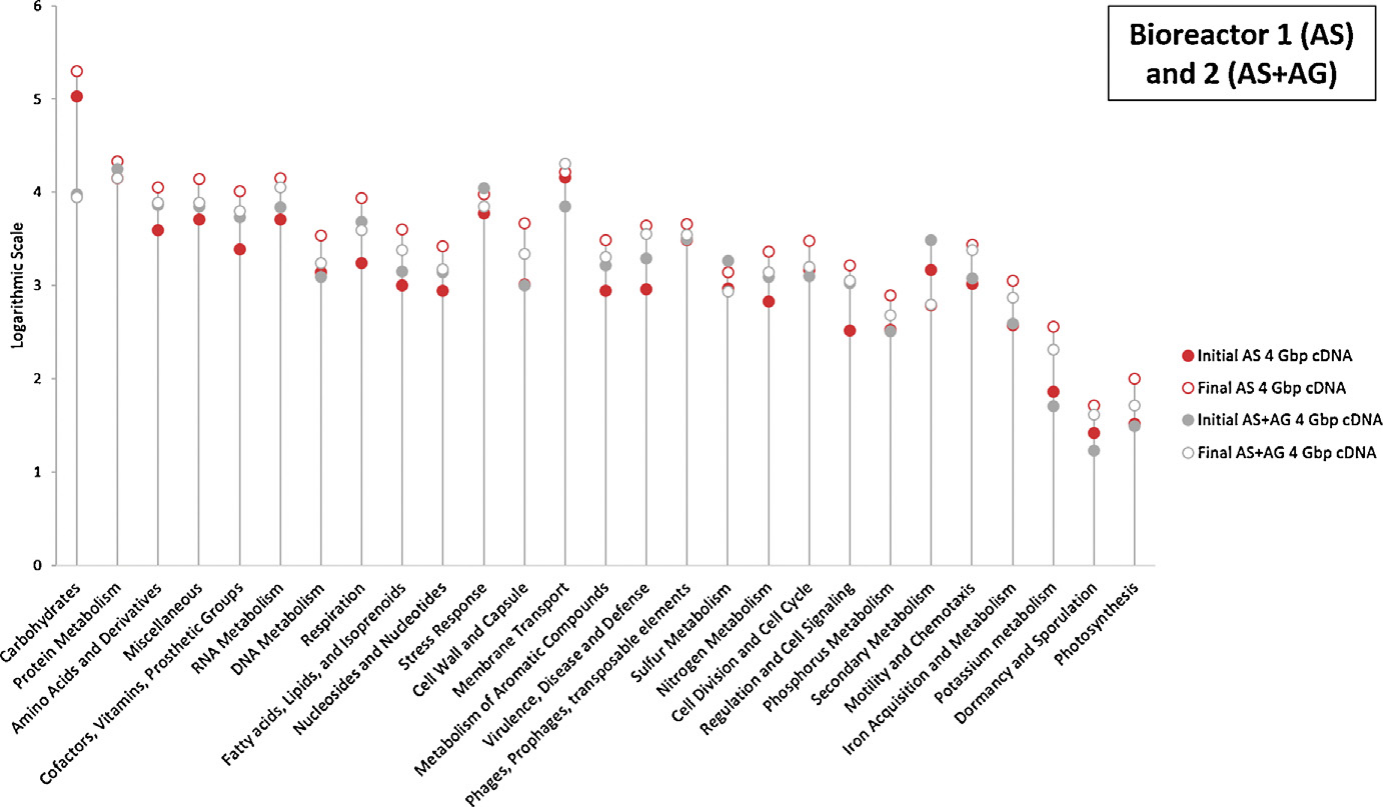
Comparison of global genes expression between metabolic systems revealed by metatranscriptomics in the Bioreactors 1 and 2, at the initial and final stages (datasets extracted from MG-RAST SEED level 1 subsystem).

When directly comparing, through metatranscriptomics, the expression rates of the different genes in the two bioreactors, as shown in Fig. 9, it appeared that, despite their similar general metabolic behaviour, the AS displayed in most of gene categories a higher final efficiency than the AS + AG, especially in the carbohydrate metabolism. Another category, crucial for removing high ammonia concentration from the WOX, is the nitrogen metabolism. Accounting for 674, 2295, 1248 and 1379 sequences for IAS_cDNA_, FAS_cDNA_, IAS + AG_cDNA_ and FAS + AG_cDNA,_ respectively, the intensity of the nitrogen metabolism clearly increased in time in B1 (AS) and was quite stable in B2 (AS + AG). However, when taking all categories into account, the nitrogen metabolism represented 0.4%, 0.7%, 1.3% and 1.2% of all sequences for IAS_cDNA_, F AS_cDNA_, IAS + AG_cDNA_ and FAS + AG_cDNA,_ respectively. This could mean that the nitrogen metabolism was proportionally more important in B2 and that the specific metabolism reactions composing the nitrogen metabolism pathway have to be weighed out regarding intensity and process efficiency.

### 3.6 Analysis of the nitrogen metabolism in gene expression

**Fig. 10:**
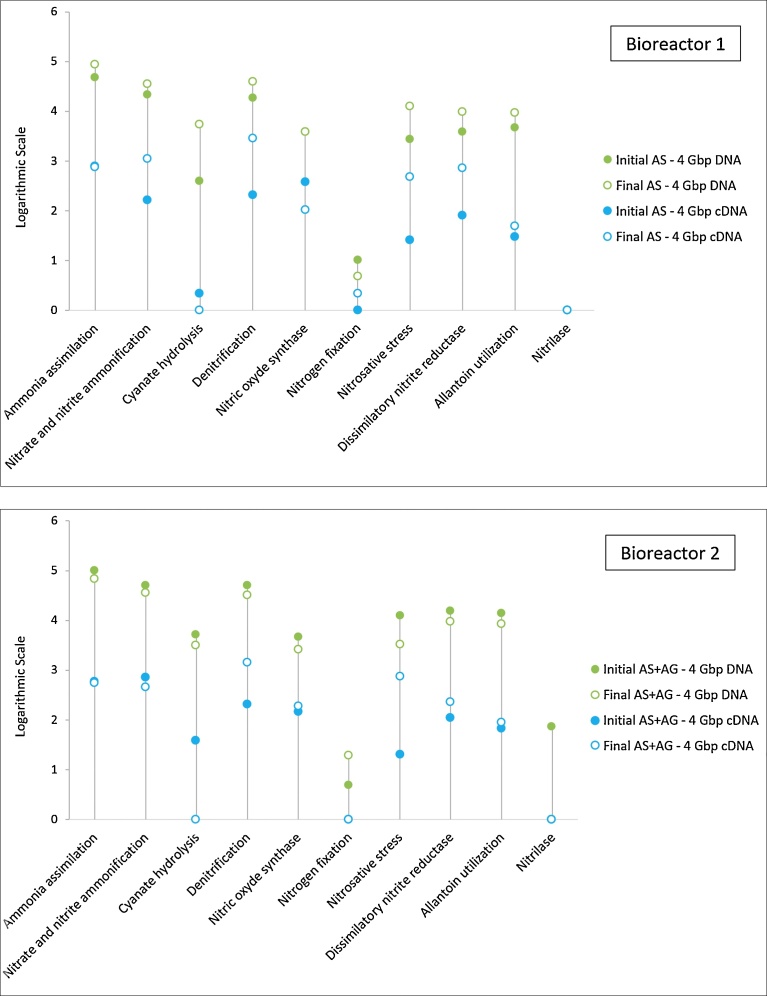
Comparison of the nitrogen metabolism genes presence and expression in Bioreactor 1 and 2, revealed by metagenomic and metatranscriptomic, at the initial and final stages (datasets extracted from MG-RAST SEED level 2 subsystem). Y-Axis scale stands for the logarithmic number of the sequences abundance (annotated sequences) per each category.

**Fig. 11:**
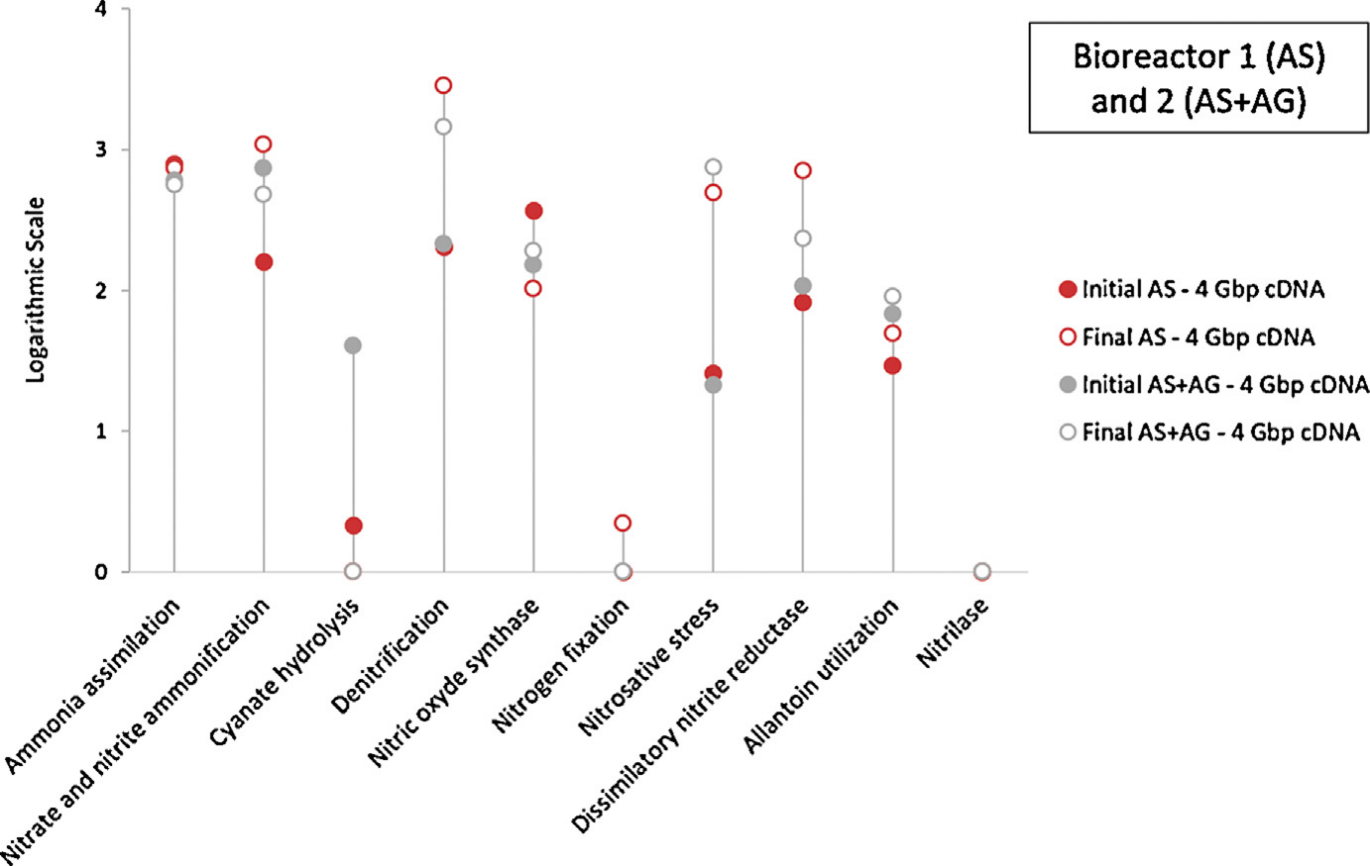
Comparison of the nitrogen metabolism genes expression in Bioreactor 1 and 2 revealed by metatranscriptomic, at the initial and final stages (datasets extracted from MG-RAST SEED level 2 subsystem).

In order to assess the metabolism effectiveness, the level 2 SEED subsystem was applied for annotation, using MG-RAST, to both assess metabolism effectiveness and characterise nitrogen metabolism processes such as ammonia assimilation, nitrite and nitrate ammonification, denitrification etc., as shown on Fig. 10 and Fig. 11. As previously described in the overall gene expression analysis, Fig. 10 confirms that, at the same sequencing depth, the abundance of annotated genes in DNA datasets (green points in Fig. 10) are generally by 1 to 2 units (based on logarithmic scale) higher than their abundance in cDNA datasets (blue points), both for bioreactors and sampling dates, and in a similar order of magnitude. If initial and final DNA datasets for all categories (green points) and for both bioreactors are always very close (genes potential), this is not the case for initial and final cDNA datasets (real genes expression), for which amplitude differences are noticeable and show how the nitrogen metabolism (blue points) evolves. Annotated genes of Ammonia assimilation (AA), followed by nitrate/nitrite ammonification (NNA) and denitrification (D) yielded the highest hit numbers in the initial and final DNA datasets for both bioreactors. When the cDNA datasets were considered, bioreactor B1 (AS), at the initial sampling time, was characterised by AA, NOS (nitric oxide synthesis), D and NNA processes, accounting for 47.5%, 22.1%, 12.3% and 9.7%, of the expressed genes of the nitrogen metabolism, respectively. After one month, the metabolic processes order changed to D, NNA, AA and DNR (dissimilatory nitrite reduction), accounting for 47.3%, 18%, 12.2% and 11.8% respectively. After one month, bioreactor B2 (AS + AG) evolved from the initial order NNA, AA, D and NOS, accounting for 37.9%, 31.1%, 11.1% and 7.7%, respectively, to D, NS (nitrosative stress), AA and NNA, accounting for 38.8%, 20%, 14.8% and 12.8% of the nitrogen metabolism genes, respectively. Thus, as confirmed in Fig. 11, both AS and AS + AG bioreactors, fed with the same stable substrate, are characterised by an increasing and strong final denitrification process, in agreement with Yu and Zhang’s results, with the highest related genes expression in AS B1. Interestingly, the Nitrosative stress process, characterised by the hydroxylamine oxidoreductase (*hao*) enzyme (which catalyses the oxidation of hydroxylamine (NH2OH) to nitrite, as part of a larger process in which ammonia is oxidised to nitrite) appeared in B2 (AS + AG) at a higher level of expression after one month, while Ammonia assimilation and Nitrite/Nitrate Ammonification decreased, confirming that the addition of Aerobic Granules contributed to the removal of ammonia.

### 3.7 Taxonomic analysis at the enzyme level of the main microbial agents involved in nitrification and denitrification

Nitrification and denitrification processes were assessed by metagenomic (taxonomic composition) and metatranscriptomic (transcriptionally active taxa) at the enzymatic level in initial and final samples. The nitrification pathway was followed by Ammonia monooxygenase (*amo*; EC 1.14.99.39), Hydroxylamine oxidase (*hao*; EC 1.7.3.4) and Hydroxylamine reductase (*har*; EC 1.7.99.1), while the denitrification pathway was assessed with Nitric-oxide reductase (*nor*; EC 1.7.2.5), Nitrous-oxide reductase (*nos*; EC 1.7.99.6), Nitrate reductase (*nar*; EC 1.7.99.4) and Nitrite reductase (NO-forming) (*nir*; EC 1.7.2.1).

**Fig. 12:**
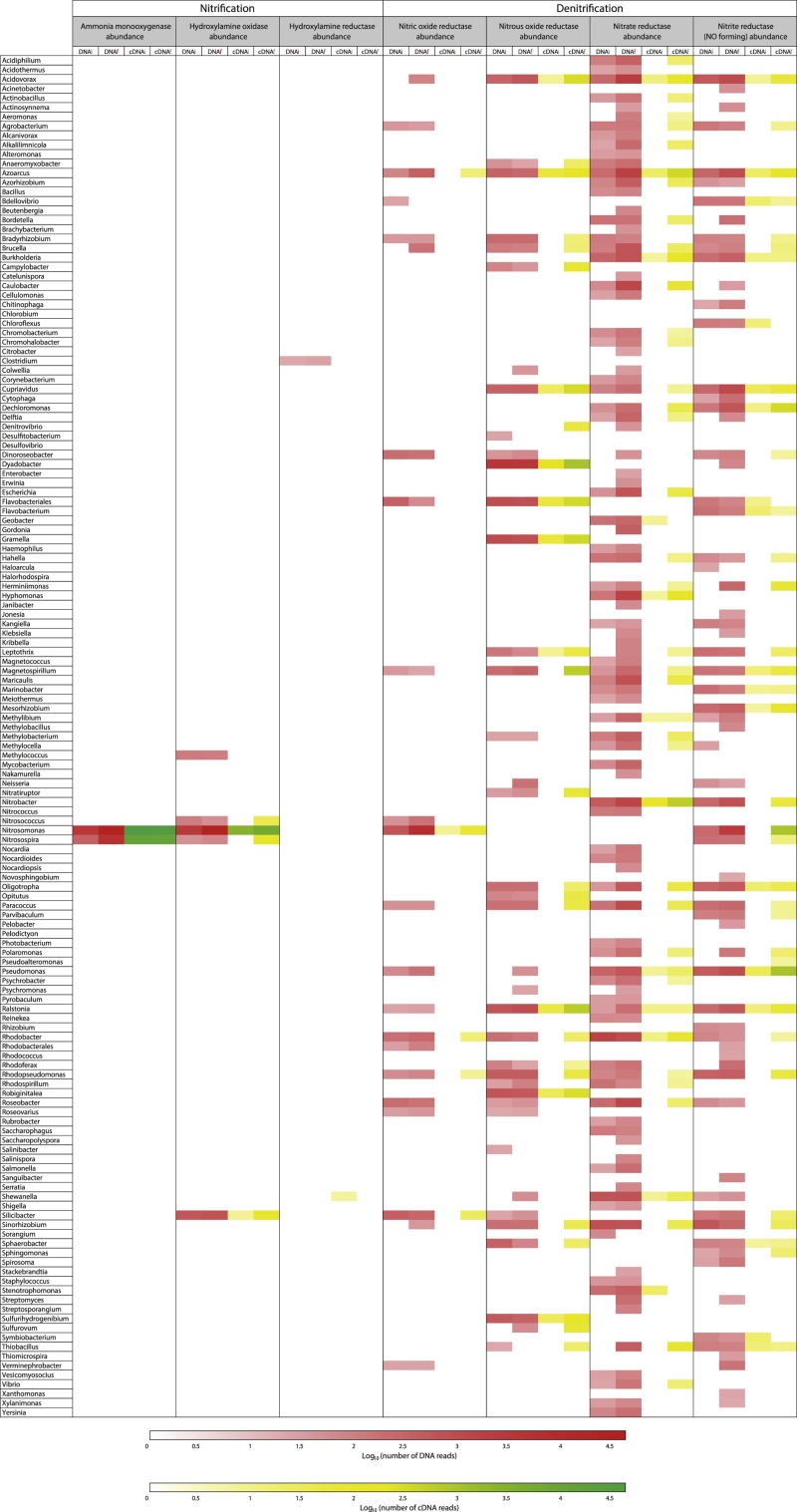
Heatmap representing the preponderant microbial genera for the genes of seven key enzymes (DNAs) of the nitrogen metabolism and their relative expression (cDNAs) at the initial and final stages, in the bioreactor 1 ‘Activated Sludge’. The abundances of genes and cDNA sequences were extracted from MG-RAST SEED level 4 (Threshold selection 85% cut off, DNA Hit ≥ 20, cDNA Hit ≥ 5).

**Fig. 13:**
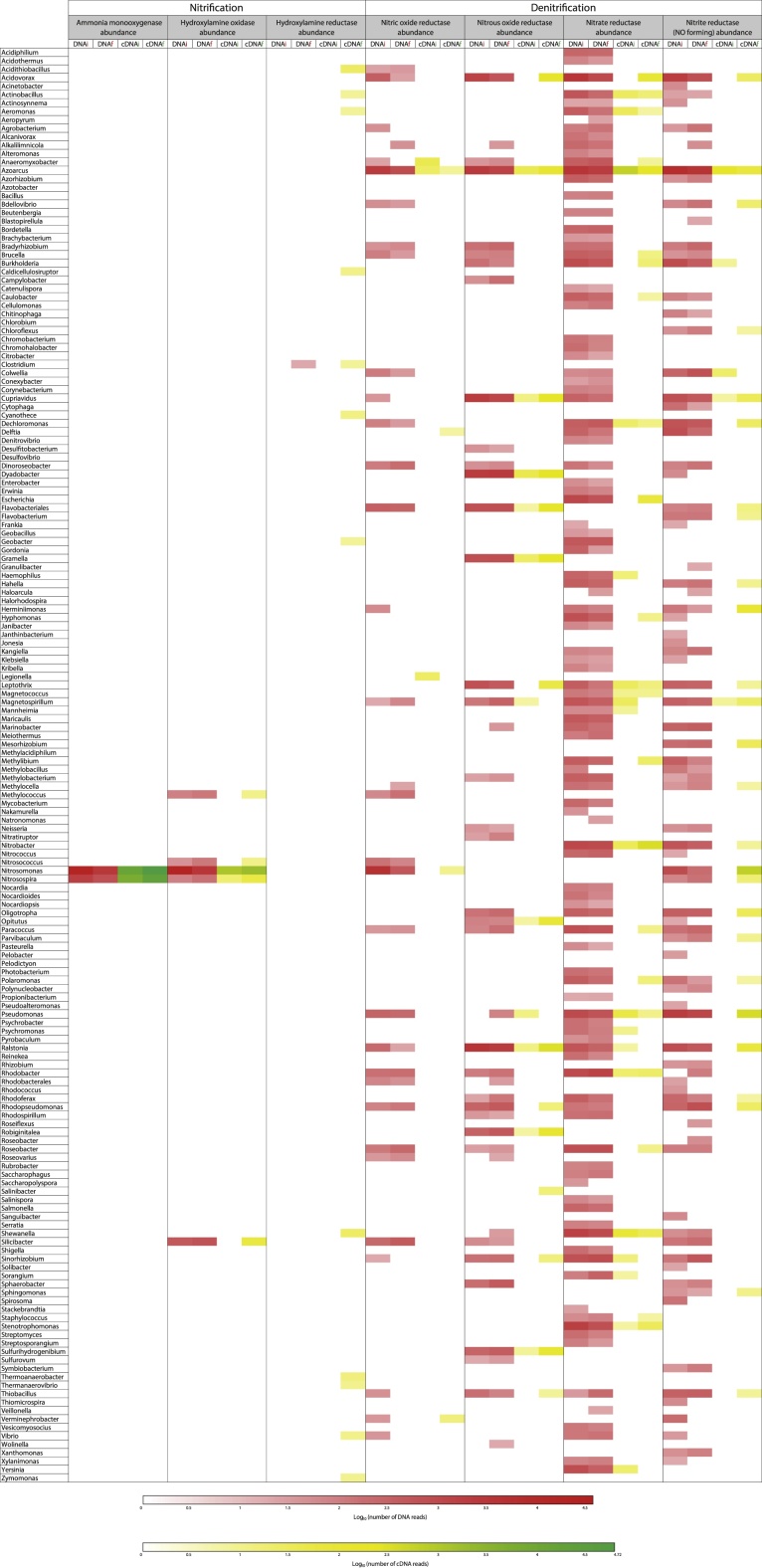
Heatmap representing the preponderant microbial genera for the genes of seven key enzymes (DNAs) of the nitrogen metabolism and their relative expression (cDNAs) at the initial and final stages in the bioreactor 2 “Activated Sludge + Aerobic Granules. The abundances of genes and cDNA sequences were extracted from MG-RAST SEED level 4 (Threshold selection 85% cut off, DNA Hit ≥ 20, cDNA Hit ≥ 5).

As described in Fig. 12 and Fig. 13, based on the SEED subsystem (85% cut off; DNA hit ≥ 20; cDNA hit ≥ 5), coding sequences of 145 and 170 bacterial genera, from B1 and B2, respectively, were annotated for at least one of these seven enzymes. A higher diversity was recovered from B2, which is congruent with the fact that aerobic granules certainly brought in an additional diversity of microorganisms. Considering only the same five enzymes of the study of Yu and Zhang (without nitrite and nitrate reductase), at the same selection thresholds, 49 and 66 different bacterial genera were annotated in B1 et B2, respectively, against 37 for Yu and Zhang’s study, with 73% of these genera being common in this latter study. Though the starting materials of both these studies were not similar, these results show a huge bacterial richness which is involved in the nitrogen metabolism, with a sequencing depth unreached so far of 4 Gb per sample, in comparison to 2.4 Gb of Yu and Zhang’s study. No *Archaeabacteria* sequences were annotated at this threshold of selection, which is in agreement with recent literature (Zhang et al., 2011; Ye and Zhang, 2011; Yu and Zhang, 2012), relating a very low participation of *Archaeabacteria* in the AS nitrification process.

Reads hits from the cDNA datasets were generally lower than reads hits from DNA datasets when recorded for the same bacterial genus, and were also characterised by a lower bacterial richness. Many enzyme coding genes were only recovered from DNA datasets for some genera, which means there was no expression of these genes.

Based on DNA datasets, for instance in the final sampling of B1 (Fig. 12), the genes *amo*, *hao*, *har*, *nor*, *nos*, *nar* and *nir* were common to 2, 5, 1, 21, 36, 96 and 66 bacterial genera respectively, and accounted for an abundance (number of occurrences of the gene) of 47821, 41522, 25, 9481, 10746, 27971 and 15516, respectively. In comparison, the same genes were common to 2, 4, 0, 5, 27, 42 and 34 bacterial genera and accounted for an abundance of 46751, 4354, 0, 92, 2755, 1323 and 1975, respectively, in cDNA datasets. This was similar in the DNA datasets of the final B2 sampling (Fig. 13), where the genes *amo*, *hao*, *har*, *nor*, *nos*, *nar* and *nir* were common to 2, 5, 1, 25, 41, 103 and 61 bacterial genera, respectively, and accounted for an abundance of 5034, 5724, 21, 4743, 18603, 29953 and 20594, respectively, whereas they were common to 2, 5, 12, 4, 15, 23 and 26 bacterial genera and accounted for an abundance of 70435, 1272, 108, 28, 953, 606 and 953 in cDNA datasets. In both bioreactors, if denitrification enzymes were shared by a wider bacterial diversity than nitrification enzymes, they displayed a weaker cumulated expression intensity.

Regarding nitrification, as shown in Fig. 12 and Fig. 13, only two bacterial genera, namely *Nitrosomonas* and *Nitrosospira,* exhibited abundance for ammonia monooxygenase in DNA and cDNA datasets of both bioreactors. The gene *amo* displayed the highest expression level per genus (cDNA characterised by yellow and green colours in the heatmaps), as well as the highest expression level among the analysed enzymes, despite the fact that it was less shared by bacterial genera than other enzyme coding genes. Moreover, considering initial and final cDNA datasets, *amo* abundance increased in B2 from 10731 to 52977 for *Nitrosomonas* and from 4102 to 17458 for *Nitrosospira*. In B1, *amo* abundance remained stable (from 38261 to 37032 for *Nitrosomonas* and from 10312 to 9719 for *Nitrosospira*). This observation supports the hypothesis that the *Nitrosomonas* genus would provide an essential share of the nitrification activity, which is enhanced by the aerated conditions of the bioreactors, this well suiting the nitrification process.

Only 5 bacterial genera were annotated for the *hao* gene in DNA and cDNA datasets of both bioreactors, namely *Methylococcus*, *Nitrosococcus*, *Nitrosomonas*, *Nitrosospira* and *Silicibacter*. In both initial and final cDNA datasets, *Nitrosomonas* by far showed the highest abundance of *hao*, which increased from 1934 to 4186 in B1 and from 707 to 1184 in B2. In comparison, *Nitrosospira* and *Silicibacter* only accounted for an abundance of 80 and 64 *hao*, respectively, in the final cDNA dataset in B1 and for an abundance of 34 and 38 *hao* in the final cDNA dataset in B2, respectively. For the hydroxylamine reductase, only one bacterial genus was listed for both bioreactors in DNA datasets, namely *Clostridium*, but surprisingly one genus was annotated in B1 and 12 genera in B2 in cDNA datasets. In this last case, the most highly abundant bacterial genus was *Acidithiobacillus* (18 hits), followed by *Shewanella* (17 hits) and *Thermoanaerobacter* (10 hits).

Regarding the denitrification process with nitric oxide reductase (*nor*), most of the annotated sequences of the final cDNA datasets belonged to *Nitrosomonas* (49 hits), *Silicibacter* (17 hits) and *Rhodobacter* (10 hits) in B1, and to *Verminephrobacter* (10 hits), *Nitrosomonas* (7 hits) and *Azoarcus* (6 hits) in B2. For nitrous oxide reductase (*nos*), most of these sequences belonged to *Dyadobacter* (612 hits), *Ralstonia* (365 hits) and *Magnetospirillum* (360 hits) in B1, and to *Ralstonia* (160 hits), *Dyadobacter* (122 hits) and *Cupriavidus* (97 hits) in B2. Another denitrification enzyme, nitrate reductase (*nar*), was characterised by the highest bacterial richness in DNA and cDNA datasets of both bioreactors. The corresponding more abundant bacterial genera, for this enzyme, in the final cDNA dataset of B1, were *Nitrobacter* (318 hits), followed by *Azoarcus* (176 hits) and *Acidovorax* (91 hits). The same genera *Nitrobacter* (183 hits) followed by *Azoarcus* (125 hits) and *Acidovorax* (57 hits), were found in the B2 dataset. Finally, the nitrite reductase (*nir*) gene displayed the second highest bacterial richness in DNA and cDNA datasets of both bioreactors, and most of the hit sequences in the final cDNA dataset were provided by *Pseudomonas* (613 hits), *Nitrosomonas* (602 hits) and *Dechloromonas* (171 hits) in B1, and by *Nitrosomonas* (365 hits), *Pseudomonas* (206 hits) and *Herminiimonas* (60 hits) in B2.

As shown in Fig. 12 and Fig. 13, even if the metagenomic and metatranscriptomic approaches appear to be complementary, metatranscriptomics seems to be more accurate for assessing the functional metabolism, allows to display the differences of transcriptional activities and differentiate living bacterial genera.

**Fig. 14:**
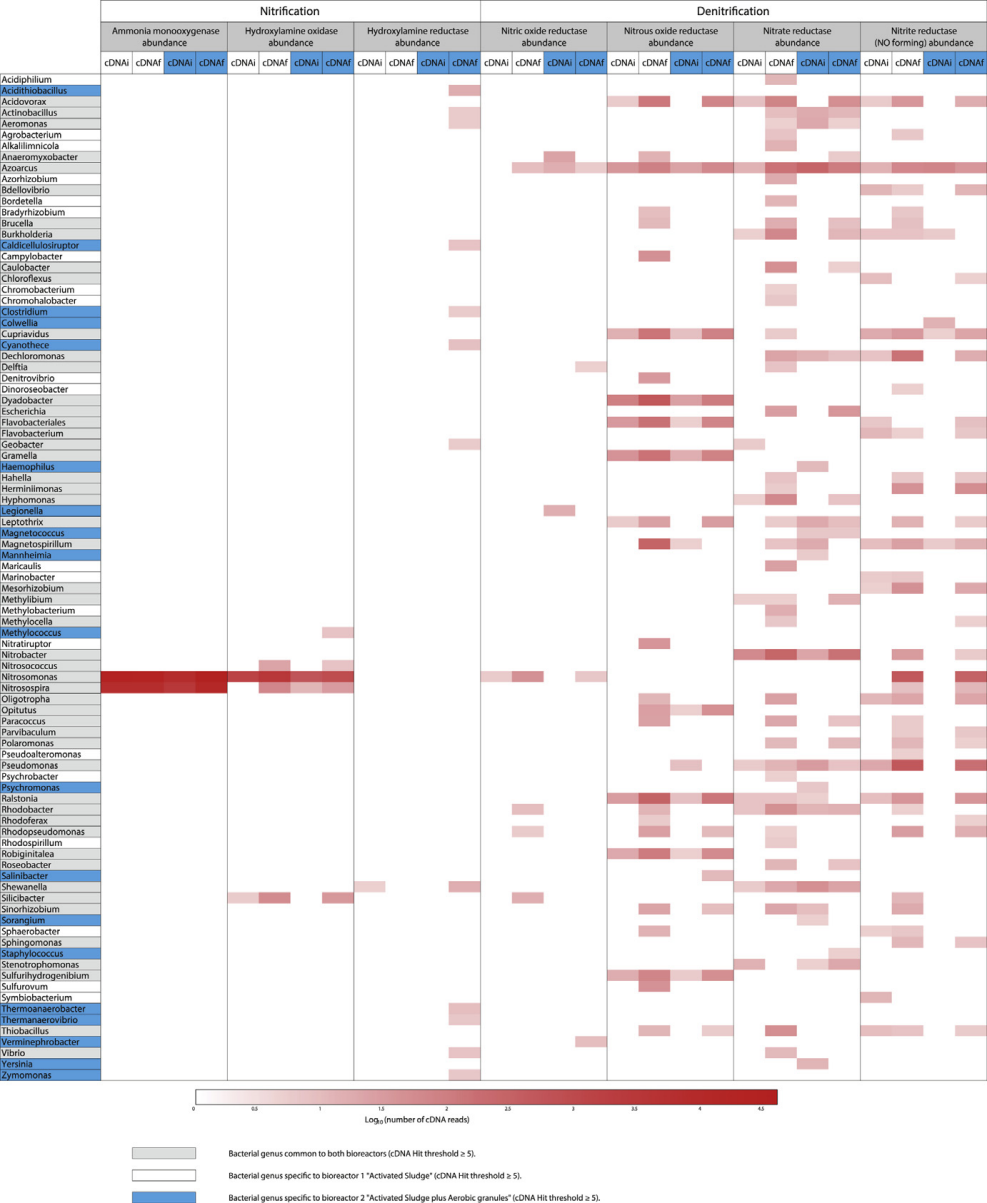
Heatmap representing the preponderant microbial genera for the relative expression of the genes of seven key enzymes (cDNAs) of the nitrogen metabolism at the initial and final stages, in the bioreactors 1 (in white) and 2 (in blue); cDNAi stands for cDNAs at the initial stage, cDNAf for cDNAs at the final stage. Genera common to the 2 bioreactors are on a grey background while genera specific to Bioreactor 1 appear on a white background and genera specific to Bioreactor 2 appear on a blue background) The abundances of genes and cDNA sequences were extracted from MG-RAST SEED level 4 (Threshold selection 85% cut off, DNA Hit ≥ 20, cDNA Hit ≥ 5).

The metatranscriptomic analysis, as described in Fig. 14, showed that B1 and B2 shared 56% (51/91) of the bacterial genera active in the nitrogen metabolism (in grey) at the chosen selection threshold (85% cut off, cDNA Hit ≥ 5 − SEED subsystem); 23% (21/91) of these genera were specific to AS B1 (in white), and 21% (19/91) were specific to AS + AG B2 (in blue). The addition of 200 mL of the AG solution to B2 clearly modified the composition of the activated sludge microbiota, which moved to a new operating balance. The nitrogen metabolism profiles of both bioreactors were found quite similar and reproducible in time, which would confirm the reliability of the metatranscriptomic analysis.

In conclusion, our works showed that, though metagenomic and metatranscriptomic approaches are complementary, metatranscriptomics allows to describe the functional metabolism and to identify predominant living bacterial genera.

The follow-up of microbial communities dynamics by metagenomics and metatranscriptomics, and particularly of the expression levels of 7 enzymes of the nitrogen metabolism showed that bacterial communities adapt to the wet oxidation effluent by increasing the expression level of the nitrogen metabolism, suggesting that these biological activities could represent a valuable alternative to reduce the cost of ammonia elimination by reducing the use of chemicals and energy consumption in sewage plants. Besides these encouraging results, this study, by reaching a high sequencing depth (from 4.4 to 7.6 Gb), enlightened a yet unknown diversity of the microorganisms involved in the nitrogen pathway and revealed the abundance and expression levels of specialised enzymes involved in nitrification, denitrification, ammonification, dissimilatory nitrate reduction to ammonium (DNRA) and nitrogen fixation processes in AS.

## Declarations

### Author contribution statement

Julien Crovadore: Conceived and designed the experiments; Performed the experiments; Analyzed and interpreted the data; Contributed reagents, materials, analysis tools or data; Wrote the paper.

Vice Soljan: Conceived and designed the experiments; Performed the experiments; Analyzed and interpreted the data; Wrote the paper.

Gautier Calmin, Romain Chablais and Bastien Cochard: Contributed reagents, materials, analysis tools or data, Analyzed and interpreted the data; Wrote the paper.

François Lefort: Conceived and designed the experiments; Analyzed and interpreted the data; Wrote the paper.

### Funding statement

This work was funded by the Federal Office for the Environment (FOEN) of the Swiss Confederation as the project “Nitrogen removal from wet oxidation" No UTF 427 23 12, and research funds of the University of Applied Sciences and Arts of Western Switzerland.

### Competing interest statement

The authors declare no conflict of interest.

### Additional information

No additional information is available for this paper.
